# Cigarette smoke affects the onco-suppressor DAB2IP expression in bronchial epithelial cells of COPD patients

**DOI:** 10.1038/s41598-019-52179-5

**Published:** 2019-10-30

**Authors:** Giulia Anzalone, Giuseppe Arcoleo, Fabio Bucchieri, Angela M. Montalbano, Roberto Marchese, Giusy D. Albano, Caterina Di Sano, Monica Moscato, Rosalia Gagliardo, Fabio L. M. Ricciardolo, Mirella Profita

**Affiliations:** 1 Institute for Biomedical Research and Innovation (IRIB), National Research Council of Italy (CNR), Palermo, Italy; 2 Dipartimento di Biomedicina sperimentale e Neuroscienze Cliniche (BioNec), University of Palermo, Palermo, Italy; 3 InterventionalPulmonology Unit, La Maddalena Cancer Center, Palermo, Italy; 4 Department of Clinical and Biological Sciences, University of Torino, Torino, Italy

**Keywords:** Inflammation, Oncogenesis

## Abstract

Cigarette smoke is a risk factor for COPD and lung cancer. In cancer, epigenetic modifications affect the expression of Enhancer of Zester Homolog 2 (EZH2), and silenced disabled homolog 2 interacting protein gene (DAB2IP) (onco-suppressor gene) by Histone H3 tri-methylation in lysine 27 (H3K27me3). *In“ex vivo”*studies, we assessed EZH2, H3K27me3 and DAB2IP immunoreactivity in bronchial epithelial cells from COPD patients (smokers, ex-smokers), Smoker and control subjects. In*“in vitro” experiments* we studied the effect of cigarette smoke extract (CSE) on EZH2/H3K27me3/DAB2IP expression, apoptosis, invasiveness, and vimentin expression in 16HBE, primary cells, and lung cancer cell lines (A549) long-term exposed to CSE. Finally, in “i*n vitro”studies*, we tested the effect of GSK343 (selective inhibitor of EZH2). EZH2 and H3K27me3 expression was higher, while DAB2IP was lower levels, in bronchial epithelium from COPD and Smokers than in Controls. CSE increased EZH2, H3K27me3 expression and decreased DAB2IP, cell apoptosis and invasiveness in epithelial cells. GSK343 restored the effects of CSE. Cigarette smoke affects EZH2 expression, and reduced DAB2IP via H3K27me3 in COPD patients. The molecular mechanisms associated with EZH2 expression, generate a dysregulation of cell apoptosis, mesenchymal transition, and cell invasiveness in bronchial epithelial cells, encouraging the progression of airway inflammation toward lung cancer in COPD patients.

## Introduction

90% of Chronic Obstructive Pulmonary Disease (COPD) are smokers or ex smokers^1^. In fact, the habit to cigarette smoke is major risk factor for COPD^1^. Inflammation of central and peripheral airways, respiratory bronchiolitis, and destruction of the lung parenchyma (as pulmonary emphysema) are pathological hallmarks of COPD^1–3^. Inflammation of the lung is strongly linked to cancer, however the mechanisms of this relationship are still unclear^4,5^.

A common environmental risk factor between lung cancer and COPD is cigarette smoke exposure. The incidence of disease, is due to a genetic predisposition, represented in a fraction of smokers. The link between cigarette smoking and lung cancer risk is well established, as well as COPD is a major independent risk factor for lung carcinoma, among the long-term exposure to cigarette smoke^5–7^. Despite the fact that tobacco carcinogens mediate inactivation of numerous tumour suppressor genes via epigenetic mechanisms, and the fact that tobacco smoke initiates and maintains the malignant phenotype of lung cancer cells, its contribution to the prognosis of lung cancer patients remain poorly understood^8^.

Enhancer of zester homolog 2 (EZH2) is the catalytic subunit of polycomb repressive complex 2 (PCR2), and its-terminal SET domain exhibits methyl transferase activity^9^. It plays an important role in epigenetic silencing of genes, by tri-methylation of Histone H3 in lysine 27 residue (H3K27me3). The target genes of EZH2 are involved in a variety of biological processes such as proliferation and apoptosis^9–13^. It is highly expressed in a wide range of cancer types, including breast, prostate, bladder, colon, lung, pancreatic cancer, sarcoma, and lymphomas^14^. In *“in vitro”* studies, EZH2 overexpression is related with increased proliferation and oncogenic capacity of cell lines^15^, while in *“ex vivo”* studies, it is related with advanced stages of human cancer progression and poor prognosis^16^.

Disabled homolog 2-interacting protein (DAB2IP), acts as a putative tumour suppressor gene, and it is downregulated by epigenetic modification in multiple aggressive cancers. DAB2IP is located at chromosome 9q33.1-q33, and it is a member of the RAS-ATPase activating protein family (RAS GAP)^17,18^. Its expression is repressed by aberrant promoter hyper methylation and histone modification in cancer (prostate, breast, lung, and gastrointestinal)^17^. The repression of DAB2IP gene controls the conveying apoptosis resistance in immortalized neural precursor and medulloblastoma cells, and in prostate cancer by polycomb EZH2 complex^17,18^.

The aim of present study is to identify the connection between the habit to cigarette smoking, chronic inflammation and tumorigenic markers studying EZH2, DAB2IP expression and H3K27me3 in an *ex vivo*/*in vitro* model of airway diseases. We first evaluated: (1)*“ex vivo* “ the EZH2, DAB2IP and H3K27me3 immunoreactivity in bronchial epithelium from COPD patients (smokers and ex-smokers), Smokers and control subjects; then we studied: (2)*“in vitro”*, the EZH2, DAB2IP andH3K27me3 expression in epithelial cells (normal and cancer cell line or primary epithelial cells) chronically exposed to Cigarette Smoke Extract (CSE). Finally, we evaluated the effect of EZH2 over-expression on cell apoptosis, epithelial to mesenchymal transition, and cell invasiveness, of epithelial cells exposed to CSE for long time.

## Materials and Methods

### Patient population

The methods for the selection of patient population was previously described^19^. Briefly, surgical specimens from tumour-free central bronchi were collected from subjects, underwent to lung resection to benign lung cancer, enrolled at the “La Maddalena Cancer Center”, Palermo. The study was approved by the ethics Committee of the Euro-Mediterranean Institute of Science and Technology of Palermo (CE/01/2017) in agreement with Helsinki Declaration. Written informed consent was obtained from each patient. We recruited four groups of subjects: COPD smokers (*n* = 10) and former smokers (*n* = 8), asymptomatic smokers with normal lung function (*n* = 8), and healthy asymptomatic nonsmoking subjects with normal lung function (*n* = 8). The diagnosis of COPD and the assessment of its severity were defined and classified according to the criteria reported by the Global Initiative for Obstructive Lung Disease (GOLD) guidelines for COPD management (GOLD stage ≥ I)^2^. COPD subjects with exacerbations within 1-month prior to the study were excluded. COPD former smoker had quit smoking for at least 1 yr. Patients with COPD and healthy smokers had a smoking history of 10 pack-year or more.

COPD patients were treated with bronchodilators and were classified based on preoperative lung function: FEV1 less than 80% of reference, FEV1/FVC less than 70%, and bronchodilation effect less than 12%. The patients were not under corticosteroid therapy (neither inhaled nor systemic) and not under antibiotics and did not have exacerbations during the month preceding the study. Subjects had negative skin tests for common allergen extracts and no history of asthma or allergic rhinitis.

### Immunohistochemistry

Tissue specimens from central bronchi were selected, and fixed with 10% neutral buffered formalin and embedded in paraffin wax. Sequential sections (3 µm thick) were placed on poly-L-lysine coated slides, deparaffinised in xylene, rehydrated in a descending ethanol series and stained with haematoxylin and eosin (HE). Immunoreactivity for EZH2, H3K27me3and DAB2IP were observed using: a mouse monoclonal Ab anti-EZH2 (clone 144CT2.1.1.5) (Thermo Fisher Scientific, Rockford, USA), Polyclonal Antibody Anti-Trimethyl-Histone H3 (Lys27) (Cat#07-449 EMD Millipore Corporation, Temecula, CA, USA) and a rabbit Polyclonal Abanti-DAB2IP (ab87811, Abcam, Cambridge, UK) in Higher airways (internal perimeter >6 mm) by Immunohistochemistry^20^. LSAB2 Dako kit (Code N° K0674) (Dako, Glostrup, Denmark) and Fuchsin Substrate-Chromogen System Dako^21^ were used for the staining. Rabbit and mouse immunoglobulins (Dako) were used for negative controls. Two independent investigators, using image analysis (Leica microscope, Wentzler, Germany) 40X magnification, evaluated sample immunoreactivity blindly. The length of the basement membrane was evaluated using a Leica Application Suite V3.3 (LAS) software (Leica) for Image Analysis. Results were expressed as the number of positive epithelial cells/mm basement membrane as previously described^22^.

### Metaplastic bronchial epithelium evaluation

We evaluated the intensity and the percentage of cell immunoreactivity for EZH2, H3K27me3 and DAB2IP in the metaplastic areas of bronchial epithelium from COPD patients (n = 8) as previously described^19^. The intensity was scored on a scale 0–3, with 0 for negative uptake, 1 for weak, 2 for moderate, and 3 for strong staining. The percentage of staining area was classified as 0 (0%); 1 (1–10%); 2 (11–50%); 3 (51–100%). The intensity and percentage score was multiplied to give a composite score 1–9 for each specimen. Composite score of 1–3 was defined as having low protein expression, and score of 4–9 was considered high expression^23,24^.

### Epithelial cell cultures

Epithelial cell cultures were performed as previously described^25,26^. Briefly, the SV40 large T antigen-transformed 16HBE cell line (16HBE), an immortalized normal bronchial epithelial cell line was used in this study. The source and origin of 16HBE was kindly provided by Dr. D. Gruenert Laboratory (University of California, San Francisco, Calif) to IBIM-CNR Italy. Furthermore, the human lung adenocarcinoma cell line (A549) provided by ATCC (Manassas, VA, USA) were used for the study. 16-HBE cells were cultured as adherent monolayers in Eagle’s minimum essential medium (MEM) supplemented with 10% heat-inactivated (56 °C, 30 min) fetal bovine serum (FBS), 1% MEM (non-essential aminoacids, Euroclone), 2 mM L-glutamine, and gentamicin (250 μg/ml). A549 cells were cultured as adherent monolayer in RPMI 1640 medium (Euroclone, Milan, Italy), supplemented as MEM used for 16HBE cell culture. 16HBE and A549 (100,000 cells/well) were plated in standard six-well culture plates (Corning Inc. Wilkes-Barre, PA) in a suitable medium and grown to confluence (70–80%) before the stimulation.

### Collection of Tissue specimens and epithelial cell processing

Tissue specimens from tumour-free central bronchi of 3 control subjects (mean age, 63 ± 3.2) were placed in a tube with sterile MEM supplemented with FBS 10%(56 °C, 30 min), 1% MEM (nonessential amino acids, EuroClone), 2 mM L-glutamine, gentamicin 500 μg/mL, and fungizone 50 mg/mL at 37 °C in water-jacketed incubator with a humidified 5% CO2 atmosphere, overnight. The day after, the tissue were cut into smaller sample sizes, and then were placed into 60-mm tissue-culture dishes coated with Collagen I, Bovine (Gibco, Waltham, MA USA) containing Bronchial Epithelial Growth Medium (BEGM, Lonza, Wokingham, UK). The primary cells derived from tissue specimens older than 6–7 days have been successfully used to generate normal human bronchial epithelial cells (NHBECs) cultures. NHBECs (200,000 cells/well) were plated in standard six-well culture plates collagen coated to proceed to stimulations, in a suitable medium at 10% FBS and grown to confluence (70–80%) before the stimulation. NHBECs were used for experiments at passage (p)1 or 2. Control experiments confirmed that there was no significant difference between the responses of the cells at p1 or p2.

### Preparation of cigarette smoke extract

Commercially available cigarettes (Marlboro Red Label, Philip Morris International, Switzerland) were used in this study. Cigarette Smoke Extract **(**CSE) was prepared as previously described with minor modifications^25^, and further diluted to the required concentration in serum-free medium. The viability of the cells exposed to CSE was evaluated by trypan blue exclusion dye assay^25^.

### Long term exposure to CSE treatment of 16-HBE, NHBECs andA549

The CSE was used to treat the cells 16-HBE, NHBECs and A549. The cells were long term exposed to CSE 10% and 20% diluted in serum-free medium (MEM or RPMI1640), for 2 hours per day, for 7, and 14 days to simulate the chronic treatment with cigarette smoke as previously described with some modifications^26^. The cells were cultured with CSE-free fresh growth medium in the presence or absence of GSK343 (1 μM) (methyltransferase EZH2 inhibitor) (Sigma, Milan, Italy)^27^. GSK343 is added to the cells 30 minutes before CSE stimulation. The viability of the cells exposed to CSE was analyzed by blue exclusion dye assay.

### Total protein extraction

Total protein extraction was performed as previously described^27^. Cells were washed with cold PBS and lysed in a buffer containing 10 mmol/L Tris-HCl (pH 7.4), 50 mmol/L NaCl, 5 mmol/L ethylene diamine tetra-acetic acid (EDTA), 1% Nonidet P-40; phosphatase inhibitors consisted of 20 mmol/L β-glycerophosphate, 0.3 mmol/L Na_3_VO_4_, and 1 mmol/L Benzamidine (ICN Biochemicals, Inc, Aurora, Ohio); and protease inhibitors consisted of complete protease inhibitor cocktail (Roche). The protein content of the supernatants was analysed using a bicinchoninic acid (BCA) assay (Pierce, Rockford, Ill); 25–30 μg of lysate was then denatured under reducing conditions by boiling for 3 min in 50 mMTris-HCl (pH 6.8), 1% sodium dodecyl sulfate (SDS), 2% β-mercaptoethanol, and 0.01% bromophenol blue. Total protein extracts were analysed by western blot.

### Western blot analysis

Western blot Analysis was performed as previously described^27^. Proteins were separated by SDS–polyacrylamide gel electrophoresis (PAGE) and transferred by electrophoresis onto Immobilon-P membranes (Millipore, Bedford, MA). After transfer, the membranes were blocked overnight at room temperature in PBS containing 3% BSA and 0.5% Tween 20 before being incubated for 1 h at room temperature with the primary Abs. After washing, the blot was incubated for 45 min with the appropriate horseradish peroxidase conjugated secondary Ab; bound Ab was detected using the ECL chemiluminescence detection system (Amersham-Pharmacia, Biotech), according to the manufacturer’s instructions. Membranes were stripped and reprobed with housekeeping proteins anti-βactin Ab to normalize differences in protein loading. (Supplementary information is available for this paper at 10.1038/s41598-019-52179-5).

### Western blot antibodies

The following antibodies were used: a mouse monoclonal Ab anti-EZH2 (clone 144CT2.1.1.5) (Thermo Fisher Scientific, Rockford, USA) (1:250), a rabbit Polyclonal anti-DAB2IP Ab (ab87811, Abcam, Cambridge, UK) (1:500), a rabbit polyclonal anti-H3K27me3Ab (Millipore, CA, USA)(1:250), a mouse monoclonal anti-β-Actin Ab (Sigma, St. Louis, MO) (1:20,000), and a mouse Monoclonal anti-Vimentin Ab (Clone Vim 3B4, Dakocytomation) (1:100).

### Gel images evaluation

Autoradiography films were scanned by means of densitometry and analyzed with Image/Gel Plotting analysis software (National Institutes of Health, Bethesda, Md) to determine band intensities. Data are expressed as arbitrary densitometric units (A.D.U.) corrected against the density of β-actin bands. The method was previously described^26^.

### Chromatin Immunoprecipitation (ChIP) assay

ChiP analysis was performed according to manufacturer’s instructions using the EZ-ChiP kit (Upstate Biotechnology,Inc., Lake Placid, NY, USA) in 16HBE cells stimulated with CSE (0% and 20%) in the presence or absence of GSK343 (1 μM) as previously described^28^. Briefly, 37% formaldehyde was added to the culture medium to immobilize the DNA–protein and protein–protein interactions. Cells were washed twice with ice-cold PBS, suspended in cell SDS lysis buffer containing protease inhibitor cocktail II and kept on ice for 15 min. Cell lysates were sonicated on ice until the cross-linked chromatin was sheared to yield DNA fragments between 200 and 1000 bp. Each immunoprecipitation was diluted 10 times with ChIP dilution buffer containing protease inhibitor cocktail II. Protein G agarose was added to each immunoprecipitated sample in order to “preclear” the chromatin and to reduce non-specific background. 50% of the supernatants were incubated overnight at 4 °C with an Anti-H3K27me3 Ab, and the other 50% served as negative (normal mouse IgG) and positive controls (anti-RNA polymerase II). After the immunoprecipitation of antibody/antigen/DNA complexes, samples were eluted and subsequently treated with 5 M NaCl at 65 °C for 5 h to reverse the crosslinks of protein/DNA complexes to free DNA. DNA purification was performed using spin columns according to the manufacturer’s instructions. Polymerase chain reaction (PCR) was performed with specific primers for 35 cycles, and amplified DNA fragments were analysed on a 2% agarose gel by electrophoresis. PCR was performed using primers spanning the H3K27me3 binding site of the human DAB2IP gene promoter: primer Rev: 5′-GGT AAC TCC CCC TCA ACG TG-3′; primer Fw: 5-CTC GCG GAG CTC AGG GGA-3. Purified DNA was then analysed by PCR using control primers specific to the GAPDH promoter, a housekeeping gene used as positive control for immunoprecipitation, in samples immunoprecipitated with anti-polimerase II.

### Human primary bronchial epithelial cell culture

The proliferated cells derived from tissue specimens have been successfully used to generate bronchial epithelial cell cultures (HBECs) as previously described^27^. When the epithelial cells reached 80–90% confluence, they were dissociated with trypsin-EDTA and passed onto plastic tissue culture plates (Corning Inc. Wilkes-Barre, PA) collagen coated to proceed to stimulations. Cells were used for experiments at passage 1 or 2. Control experiments confirmed that there was no significant difference between the responses of the cells at passage 1 or 2.

### RNA isolation and qRT-PCR

Total RNA was extracted from cell lines with TRIzol Reagent (Invitrogen, CA, USA) following the manufacturer’s instructions, and was reverse-transcribed into cDNA, using M-MLV-RT and oligo(dT) primer (Invitrogen, CA, USA). Quantitative real-time PCR of DAB2IP transcript was carried out on StepOnePlus Real-time PCR System (Applied Biosystems, Foster City, CA, USA) using specific FAM-labelled probe and primers (revalidated TaqMan Gene expression assay for DAB2IP Hs00368995, Assays on Demand, Applied Biosystems). DAB2IP gene expression was normalized to glyceraldehyde-3-phosphate dehydrogenase (GAPDH) endogenous control gene. Relative quantitation of gene expression was carried out with the comparative C_T_ method (2^−ΔΔCt^) and was plotted as fold-change compared to untreated cells chosen as the reference sample.

### Cell apoptosis by flow cytometry

The cells A549 were stained with a solution containing a mixture of Annexin V-FITC in binding buffer 1X a previously described^29^. After incubation (15 min in total darkness, RT) was add Propidium Iodide just before analysis. The number of viable, apoptotic and necrotic cells were determined using the FACS Calibur flow cytometer (Becton Dickinson, San Jose, CA). Results were presented as a percentage of counted cells.

### Matrigel invasion assay

BioCoatMatrigel Invasion Chamber (BD Biosciences-Discovery Labware, Inc Bedford, MA) was used to measure cell invasion according to manufacturer’s instruction. Briefly, A549 cells were stimulated with chronic CSE alone or in combination with GSK343 as described above. After 14 days of stimulation, cells were allowed to invade for 22 hours toward 5% FBS. The cells on the upper surface of the matrigel were removed, fixed with methanol and the membranes stained with Diff quick, and the cells adherent to the outer surface of membrane evaluated, counting at least six fields per filter in each group at 40X magnification.

### Double indirect immunofluorescence of A549 cells line

After 14 day of stimulation with CSE (10% and 20%) in the presence or absence of GSK343 (1 μM), A549 cells were detached with trypsin and seeded on top of a sterile coverslip that was lying inside each well of a six well plate. Cells were grown until 70–80% of confluence and treated for the immunofluorescence analysis s previously described^19^. After incubation, the coverslip containing the cells were washed with PBS, fixed in Paraformaldehyde 4% for 15 min RT, then washed in PBS and permeabilized for 5 min with Buffer (0.05% Saponin in PBS- plus 3% BSA) and then incubated with blocking Solution (BSA 0,5% in PBS) 1 h RT. Immunofluorescent staining was performed with a Mouse Monoclonal anti-Vimentin Ab (Clone Vim 3B4, Dakocytomation) 1:100 1 h RT followed by a secondary anti Mouse IgG (whole Molecule) Ab R-Phycoeritrin conjugate (P9287 Sigma) in the dark for 1 hour at RT. After washes in PBS, slides were counterstained with Hoechst 33342 (Sigma-Aldrich, Inc, Milan, Italy) 1:1000 in PBS for 10 min at RT and washed again in PBS. The slides were mounted Vectashield (Vector Laboratories, Burlingame, CA). Images were analysed by using a laser-scanning microscope ZEISS at a final magnification of 40X.

### Statistical analysis

Kolmogorov–Smirnov Normality test was performed to assess normal data distribution. Comparisons between groups of patients were done by unpaired student’s t test or Mann-Whitney U test for normally and non-normally distributed variables, respectively. *In vitro* experiments were normally distributed and analyzed using ANOVA, followed by Fisher’s correction. Data were expressed as mean ± S.D. All statistical analyses were performed using StatView® 5 software (SAS institute Inc). A p valueless than 0.05 was considered statistically significant in these analyses.

## Results

### Demographics characteristics of the subjects

The demographic characteristics and the functional evaluations of the studied groups are shown in Table 1. All recruited patient groups were similar with regard to age.

**Table 1:** Data are shown as mean ± S.D. Abbreviations: Controls = healthy asymptomatic non-smoking subjects with normal lung function; COPD = patients with chronic obstructive pulmonary disease; FEV1 = forced expiratory volume in 1 s; FVC = forced vital capacity.

Subject number	Controls	Smokers	COPD	COPD-Smokers
	(n = 8)	(n = 8)	(n = 8)	(n = 10)
Gender (M/F)	4/4	3/5	6/2	5/5
Age (years)	61 ± 10	60 ± 20	67 ± 2	69 ± 2
Pack/Years	—	55 ± 8	53 ± 33	53 ± 5
FEV1% of predicted	90 ± 15	97 ± 8	69 ± 15	62 ± 6
FEV1/FVC %	89 ± 8	96 ± 7	63 ± 7	69 ± 7

### EZH2 and DAB2IP expression in COPD patients and Control subjects

**Figure 1:**
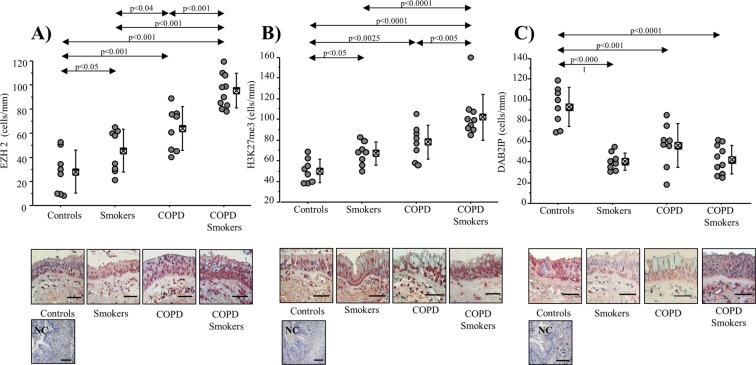
Immunoreactivity for EZH2, H3K27me3 and DAB2IP in bronchial epithelial cells (cell/mm) from Control (*n* = 8), Smokers (n = 8), COPD ex-smoker (*n* = 8) and COPD smoker (*n* = 10) subjects. Cells were stained with the following antibodies: (**A**) anti-EZH2, (**B**) anti-H3K27me3; (**C**) anti-DAB2IP. Negative Control (NC) was performed using rabbit immunoglobulins. The scale bar is 50 µm. Counts for the number of positive epithelial cells/mm, basement membrane are shown. Bars represent mean ± SD of positive bronchial epithelial cells/mm basement membranes. Representative Immunostaining of EZH2, H3K27me3 and DAB2IP in Control (*n* = 8), Smoker (*n* = 8), COPD ex-smokers (*n* = 8) and COPD Smokers (*n* = 10) subjects at 40× magnification is shown. Statistical analysis was performed by Student’s t-test. Significance was accepted at *p* < 0.05.

We analyzed EZH2, H3K27me3 and DAB2IP immunoreactivity in bronchial epithelial cells (cells/mm) from section of surgical specimens of COPD (smokers and ex-smokers) patients, and of Control subjects by immunohistochemistry. The epithelial cells expressing EZH2 and H3K27me3 were significantly increased in COPD (smokers and ex-smokers), and in Smoker subjects (p < 0.05, p < 0. 001 and p < 0.001, respectively; p < 0.05, p < 0. 0025 and p < 0.0001, respectively) compared to Control subjects (Fig. 1A,B). EZH2 immunoreactivity significantly increased in bronchial epithelium of COPD Smokers compared to Smokers subjects and to COPD ex-smokers (p < 0.001 and p < 0.001, respectively) and in COPD ex-smokers compared to Smokers (p < 0.04). Furthermore, H3K27me3 immunoreactivity significantly increased in bronchial epithelium of COPD smokers compared to smokers and to COPD ex-smokers (p < 0.0001 and p < 0.005; respectively). Conversely, the expression of the onco-suppressor DAB2IP significantly decreased in the epithelium of COPD (smokers and ex-smokers) patients, and of Smokers subjects (p < 0.0001, p < 0. 001 and p < 0.0001, respectively) in comparison to Control subjects (Fig. 1C).

### EZH2 and DAB2IP expressions in the area of metaplastic epithelium of COPD patients

**Figure 2:**
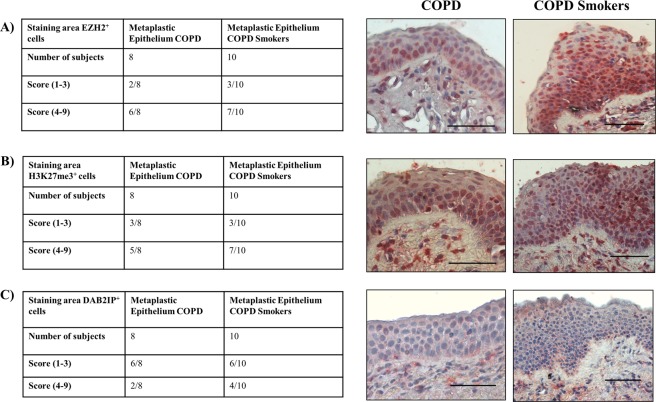
Immunoreactivityfor EZH2, H3K27me3 and DAB2IP in the bronchial epithelial cells from the area of metaplasia with weak staining (Score 1–3), and strong staining (Score 4–9) from central airways of COPD (Smokers and ex- smokers) patients. (**A**) EZH2 (n = 8); (**B**) H3K27me3 (n = 8); (**C**) DAB2IP (n = 8). Cells were stained with specific antibodies (see Materials and methods for details). Negative Control (NC) was performed using rabbit immunoglobulins. The scale bar is 50 µm. The intensity and percentage scores were multiplied to give a composite score of [1–9] (see Materials and methods for details). Representative Immunostaining at 40× magnification is shown.

The analysis of EZH2, H3K27me3 andDAB2IP immunoreactivity (cells/mm) revealed different levels of expression (in term of score) in the area of metaplastic epithelium of COPD ex-smokers (n = 8) patients. 6/8 had a higher score of EZH2 expression (Fig. 2A), 5/8 patients had a higher score of H3K27me3 (Fig. 2B), and conversely, only 2/8 COPD patients had a higher score of DAB2IP (Fig. 2C).Furthermore, in the area of metaplastic epithelium of COPD Smokers (n = 10) patients, 7/10 COPD smokers had a higher score of EZH2 expression (Fig. 2A), 7/10 patients had a higher score of H3K27me3 (Fig. 2B), and conversely, only 4/8 had a higher score of DAB2IP (Fig. 2C).

### CSE increased EZH2 and reduced DAB2IP expression in 16HBE cells

**Figure 3:**
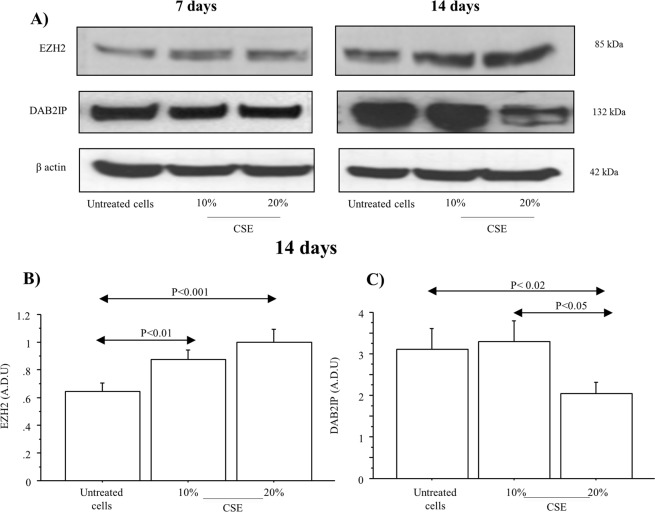
EZH2 and DAB2IP protein expression in 16HBE cells exposed to chronic stimulation with CSE. 16HBE were stimulated with/without CSE (10 and 20%) for 7 (n = 3), and 14 (n = 3). (**A**) Representative Western blot analyses of EZH2, DAB2IP, and β-actin after 7, and 14 days of stimulation with CSE (10 and 20%). (**B**) Densitometric analysis of EZH2 (n = 3); (**C**) of DAB2IP (n = 3) after 14 days of treatment with/without CSE (10 and 20%). Bars represent mean ± SD of arbitrary densitometry units (A.D.U.), normalized to β-actin used as the loading control. Statistical analysis was performed by ANOVA test with Fisher’s correction for multiple comparisons. Significance was accepted at *p* < 0.05.

The chronic stimulation of 16HBE cells with CSE (10 and 20%) induced an overexpression of EZH2 after 7, and 14 days of treatment, and conversely a downregulation of DAB2IP, as shown by the selected representative western blot images (Fig. 3A). The densitometric data shown a significant increase of EZH2 expression in 16HBE cells treated with CSE (10% and 20%) for 7 and 14 days compared to untreated cells (p < 0.01 and p < 0.001, respectively) (Fig. 3B). Conversely, a significant decrease of DAB2IP expression was observed in the cells stimulated with 20% of CSE compared to untreated cells or to the cells stimulated with 10% of CSE (p < 0.02 and p < 0.05, respectively) (Fig. 3C).

### H3K27me3 expression and its association to the promoter of DAB2IP gene in 16HBE cells

**Figure 4:**
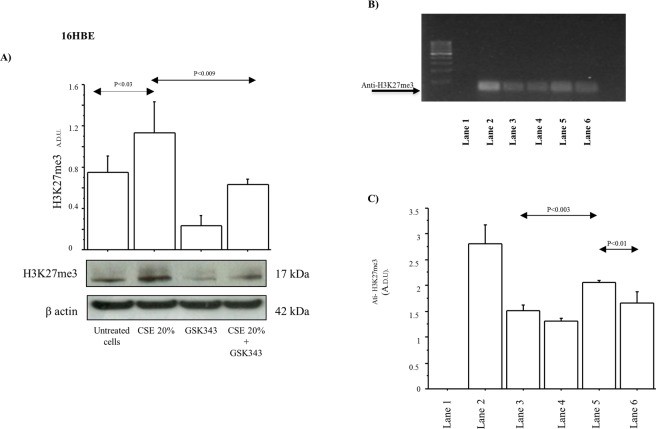
Effect of CSE and GSK343 on H3K27me3 protein expression and on the H3K27me bound to the DAB2IP promoter in 16HBE cell line. (**A**) 16HBE cells were stimulated with CSE (10–20%) for 14 days w/wo GSK343 and analysed by Western blot (n = 3). Bars represent mean ± SD of arbitrary densitometry units (A.D.U.), normalized to β-actin used as the loading control. Representative Western blot analyses of H3K27me3, and β-actin are shown. Statistical analysis was performed by ANOVA test with Fisher’s correction for multiple comparisons. Significance was accepted at *p* < 0.05. (**B**) Chromatin Immunoprecipitation (ChIP) of H3K27me3 present on the promoter of DAB2IP gene in 16HBE cells. Cells were stimulated with CSE (20%) alone or in combination with GSK343, for 14 days and then ChiP assay was performed using an Anti-H3K27me3 Ab, and PCR was performed using primers spanning the DAB2IP binding site of the human gene promoter. Lane 1, negative control of PCR; lane 2, positive control of PCR; lane 3, untreated cells; lane 4, GSK343; lane 5, CSE 20%; lane 6, CSE 20% + GSK343. Purified DNA was analyzed by PCR using control primers specific for the GAPDH promoter. Representative panel of *n* = 3 experime*n*ts was shown; (**C**) Bars represent mean ± SD of arbitrary densitometry units (A.D.U.) of ChiP Assays.

The analysis of protein extracts showed a significant increase of H3K27me3 expression in 16HBE cells stimulated with CSE (10% and 20%) for 14 days compared to untreated cells, and the treatment of the cells with EZH2 inhibitor GSK343 significantly reduced the levels of H3K27me3 expression in the cells stimulated with CSE (Fig. 4A). Chip technique was performed to further investigate epigenetic phenomena associated with EZH2-mediated repression of DAB2IP gene through H3K27me3. The results of ChIP assays showed that H3K27me3 Ab was associated to the promoter region of DAB2IP in 16HBE cells stimulated with CSE 20%, and the pretreatment of the cells with GSK343 (1 μM) reduced the intensity of the bound (Fig. 4B,C).

### DAB2IP and H3K27me3 protein expression in NHBECs

**Figure 5:**
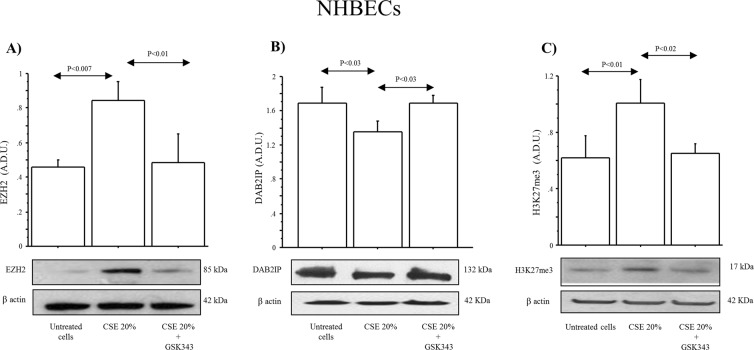
Effect of CSE and GSK343 on DAB2IP and H3K27me3 expression in NHBECs (n = 3).NHBECs were stimulated with CSE alone and in combination with GSK343 to evaluate (**A**) EZH2, (**B**) DAB2IP, (**C**) H3K27me3 expression by Western blot. Bars represent mean ± SD of arbitrary densitometry units (A.D.U.), normalized to β-actin used as the loading control. Representative Western blot analyses of DAB2IP, EZH2, H3K27me3, and β-actin are shown. Statistical analysis was performed by ANOVA test with Fisher’s correction for multiple comparisons. Significance was accepted at *p* < 0.05.

NHBECs stimulated with CSE 20% for 14 days showed a significant increase of EZH2 protein compared to untreated cells (p < 0.03), and its expression was reduced when the cells were stimulated with CSE in the presence of GSK343 (p < 0.007) (Fig. 5A). Furthermore we observed significant lower levels of DAB2IP together with higher levels of H3K27me3 in NHBECs stimulated with CSE 20% compared to untreated cells (p < 0.03 and p < 0.01, respectively). Again, the treatment of the cells with GSK343 restored the basal values of DAB2IP and of H3K27me3in the cells stimulated with CSE (p < 0.02) (Fig. 5B,C).

### Levels of DAB2IP mRNA are differentially expressed in 16HBE, and A549cell line

**Figure 6:**
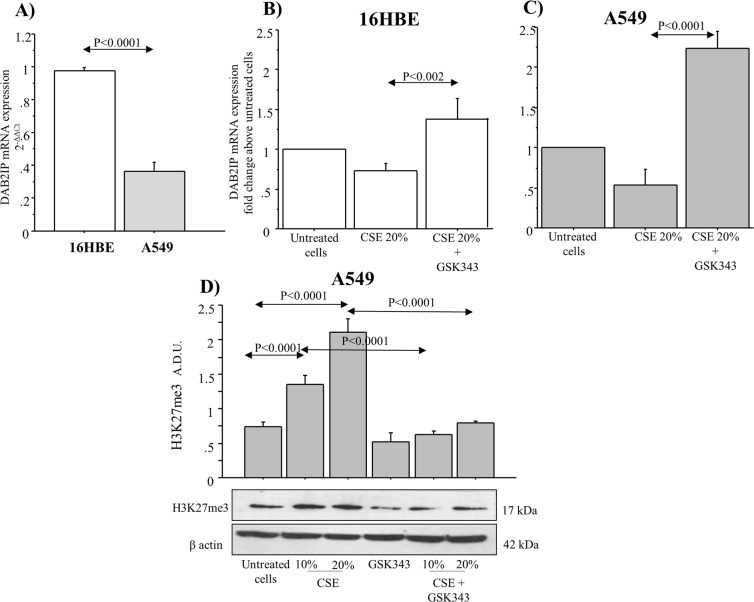
Comparison of the levels of DAB2IP mRNA expression in lung cell lines. (**A**) Basal expression levels of DAB2IP mRNA in 16HBE, and A549 cell lines (n = 3). Bars represent mean ± SD of 2^-ΔΔCT. DAB2IP mRNA expression. (**B**) 16HBE and (**C**) A549 stimulated with CSE w/wo GSK343for 14 days (n = 3). Bars show the levels of DAB2IP mRNA expressed as fold-change compared to untreated cells chosen as the reference sample. (**D**) Expression of H3K27me3 in A549 stimulated with CSE (10 and 20%) for 14 w/wo GSK343 8n = 3). Bars represent mean ± SD of arbitrary densitometric units (A.D.U.), normalized to β-actin used as the loading control. Representative Western blot analyses of H3K27me3 and β-actin was shown. Statistical analysis was performed by ANOVA test with Fisher’s correction for multiple comparisons. Significance was accepted at *p* < 0.05.

DAB2IP mRNA**(**2^−ΔΔCt^) showed lower levels in A549 than in 16HBE cell line (p < 0.0001) (Fig. 6A). The stimulation of 16HBE, and A549 cells with CSE 20% for 14 days reduced the levels of DAB2IP mRNA (fold change above untreated cells) in comparison to untreated cells. Cells stimulated with CSE in the presence of GSK343 inhibitor significantly increased DAB2IP mRNA in comparison to the cells treated with CSE 20% alone. The increase was higher in A549 cancer cell lines than in 16HBE when compared with untreated cells (p < 0.0001 and p < 0.002 respectively) (Fig. 6B,C). Additionally, we observed that the stimulation of A549 with CSE (10 and 20%) for 14 days significantly increased H3K27me3 in comparison to untreated cells (p < 0.0001, and p < 0.0001, respectively). The treatment of the cells with GSK343 reduced H3K27me3 in untreated cells and in the cells treated with CSE (p < 0.0001, and p < 0.0001, respectively) (Fig. 6D).

### Apoptosis, invasion and vimentin expression in A549 cell line stimulated with CSE

**Figure 7:**
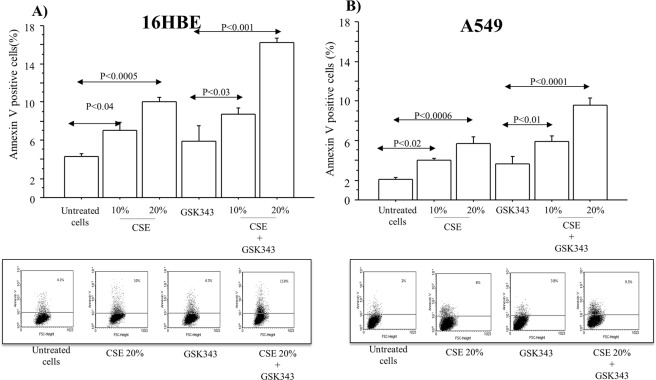
Effect of CSE and GSK343 on the apoptosis of 16HBE and A549 cell lines. (**A**) 16HBE and (**B**) A549 were stimulated with CSE (10–20%) w/wo GSK343 for 14 days (n = 3) and analysed for Annexin V by flowcytometry. Bars represent mean ± SD of percentage of Annexin V positive cells (n = 3). Representative Flowcytometry of apoptosis was shown. Statistical analysis was performed by ANOVA test with Fisher’s correction for multiple comparisons. Significance was accepted at *p* < 0.05.

**Figure 8:**
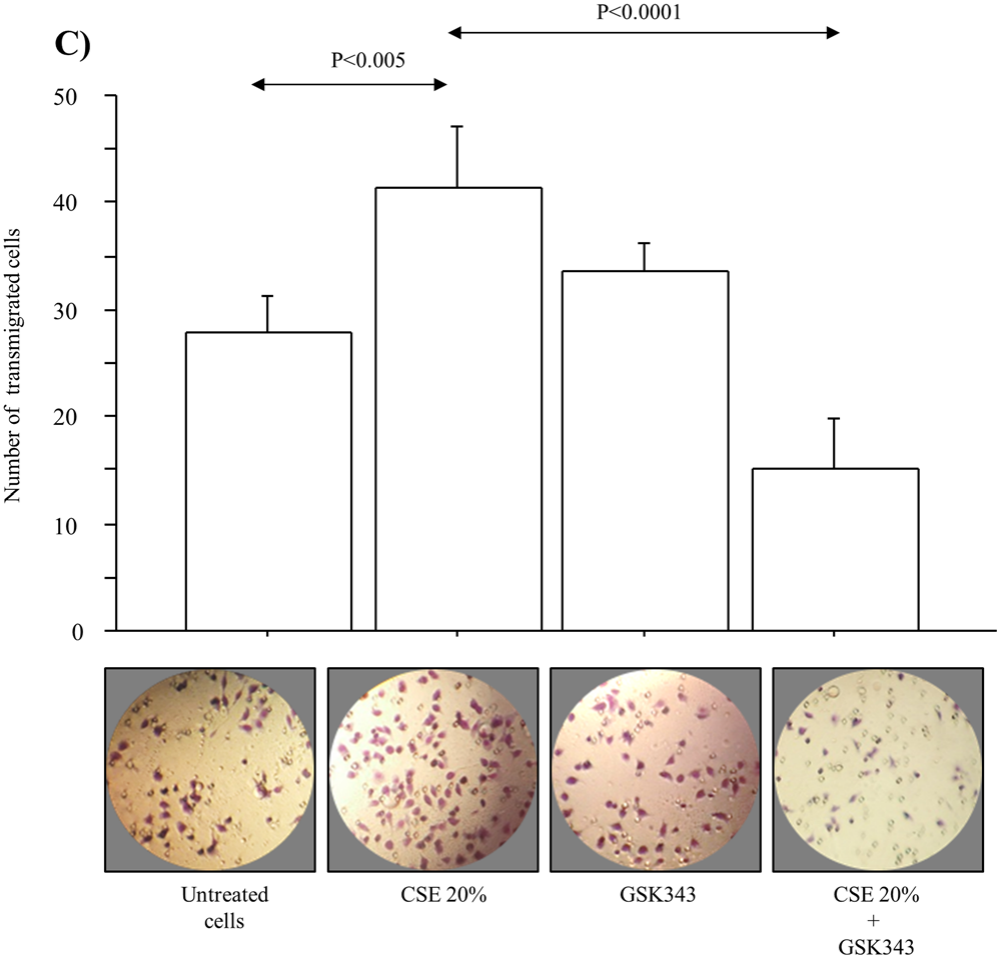
Effect of CSE and GSK343 on invasion of A549 cell line. A549were stimulated with CSE (10–20%) w/wo GSK343 for 14 days (n = 3). Cells were seeded on Matrigel-coated membrane for 22 hours, fixed and stained with Diff quick to assay invasion (n = 4). Bars represent the mean ± SD of the number of A549 cells invaded through Matrigel-coated membrane. Representative Diff Quick of the membrane at 40× magnification are shown. Statistical analysis was performed by ANOVA test with Fisher’s correction for multiple comparisons. Significance was accepted at *p* < 0.05.

**Figure 9:**
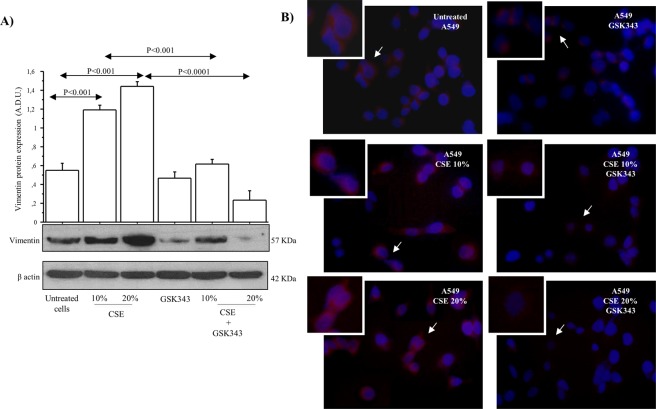
Effect of CSE and EZH2 inhibitor GSK343 on Vimentin protein expression in A549 cells stimulated with chronic CSE for 14 days. (**A**) Vimentin analysis was performed by Western blot in A549 cell stimulated for 14 days with CSE 10–20% alone and in combination with GSK343(n = 3). Bars represent mean ± SD of arbitrary densitometric units (A.D.U.) normalized to β-actin. Representative Western blot analyses of Vimentin expression, and β-actin are shown. Statistical analysis was performed by ANOVA test with Fisher’s correction for multiple comparisons. Significance was accepted at *p* < 0.05. (**B**) Representative Vimentin expression in A549 cells stimulated with CSE (10–20%) w/wo GSK343 for 14 days (n = 3) by immunofluorescence analysis.

Both, 16HBE and A549 cells stimulated with CSE 10% and 20% showed a significant increase of Annexin V-positive cells (%) compared to untreated cells (p < 0.04 and 0.0005, respectively in 16HBE; p < 0.02 and 0.0006, respectively in A549). The pre-treatment of16HBE and A549 stimulated with CSE 10% and 20%with GSK343 significantly increased the Annexin V-positive cells (%) in comparison to the cells treated with GSK343 alone (p < 0.03 and p < 0.001, respectively in 16HBE; p < 0.01 and p < 0.0001, respectively in A549)(Fig. 7). Finally we showed that, CSE 20%significantly increased the invasion capacity of A549 cell line compared to untreated cells (p < 0.005). The use of EZH2 inhibitor (GSK343) in the experimental conditions, significantly reduced the invasion capacity of A549 cells stimulated with CSE (p < 0.0001) (Fig. 8) suggesting the potential role of EZH2 in the EMT transition. Furthermore, Vimentin expression significantly increased in A549 cells stimulated with CSE (10–20%) for 14 days (p < 0.001 and p < 0.001, respectively) in comparison to untreated cells, and was significantly reduced in the cells stimulated with CSE (10–20%) in the presence of GSK343 (EZH2 inhibitor),(p < 0.001 and p < 0.0001, respectively) (Fig. 9A). Accordingly, the study of Vimentin expression by immunofluorescence confirmed the results obtained by western blot (Fig. 9B).

## Discussion

Our study showed that the habit to the cigarette smoke can induced the epigenetic modifications of DAB2IP onco-gene, via the EZH2-mediated H3K27me3 activity, in bronchial epithelial cells during the chronic inflammatory process of the airways. These modifications are present in COPD patients and smoking control subjects who smoke, and are persistent in COPD patients how have stopped smoking. The impaired expression of these markers might generate the abnormal cell proliferation in the area of metaplastic epithelium, suggesting a potential involvement of EZH2/H3K27me3/DAB2IP pathway in the progression of inflammatory diseases of the airways toward lung cancer.

The epithelium of bronchi orchestrate the inflammatory responses seen in COPD^30^. Alterations of repair mechanisms, generation of squamous cell metaplasia, and of goblet and basal cell hyperplasia are present in the airway epithelium of smokers with and without COPD, causing airflow limitation^31–33^. They are pre-invasive markers for lesions of lung cancer^34,35^. In the last years, the molecular mechanisms of this process, and epigenetic events are linked with modifications of the chromatin structure and methylation of gene promoter^7,36^. Human methyl-transferase EZH2is severally over-expressed in different types of cancer. It regulates the transcription repression of a large number of onco-suppressor genes, like DAB2IP in a variety of tumors^37–39^. CSE affects the epigenetic silencing of microRNA-218 via EZH2-mediated H3K27 trimethylation, promoting the malignant transformation of HBE cells^40^. DAB2IP is a scaffold protein involved in the regulation of cell proliferation, survival and apoptotic pathways^38^. Its down-regulation promotes mesenchymal-to-neuro-epithelial transition and neuronal differentiation of human mesenchymal stem cells^41^.

We detected higher levels of EZH2 and H3K27me3, as well as lower levels of DAB2IP expression in bronchial epithelial cells of COPD patients (smokers and ex-smokers) and Smokers than in control subjects. Furthermore, the area of metaplastic epithelium of COPD (smokers and ex-smokers) patients showed high score of EZH2 and H3K27me3 immunoreactivity, as well as low score of DAB2IP immunoreactivity. Our findings suggest that the altered EZH2/ H3K27me3/DAB2IP expression observed in COPD patients could be cause of an irregular proliferation in epithelial cells, present in the area of the metaplasia, known to be a pre-neoplastic change in response to toxic injury induced by cigarette smoke^5^. In this scenario we concluded that the habits to cigarette smoke might promote epigenetic modifications of EZH2/H3K27me3/DAB2IP, which the onset of pathogenic mechanisms of COPD, persist even after smoking cessation, encouraging the progression of inflammation toward lung cancer. Finally, we observed similar levels of DAB2IP immunoreactivity in bronchial epithelial cells of smokers, COPD ex-smokers and COPD smokers, while the levels of EZH2 and H3K27me3 increase for all these patient groups. These data might suggest further study, to understand whether the increased levels of EZH2 and H3K27me3 generate other epigenetic modifications in addition to the altered expression of onco-suppressor DAB2IP in all group of patients.

*In vitro* cell culture models are an invaluable model for understanding the change of physiological properties due to interaction between environmental/inflammatory stimuli and human airway epithelium. We used a model of chronic exposure to study the effect of cigarette smoke in bronchial epithelial cell line 16HBE. Long-term exposure to CSE show increased levels of EZH2 and H3K27me3 in 16HBE, as well as a massive decrease of the onco-suppressor DAB2IP protein, compared to untreated cells. A limited number of experiments were performed on NHBECs (obtained from surgical specimens) to support data obtained using 16HBE cells. The difficulties associated with technical procedures to separate NHBE from surgical specimens, led us to exclude the condition with GSK343 alone in the experiments. Our ChIP assay identify higher levels of H3K27me3 associated with the region of DAB2IP promoter, in 16HBE chronically exposed to CSE in comparison to untreated cells. GSK343 dow-regulated the activity of H3K27me3 in both experimental conditions. In this manner we showed a direct transcriptional suppression of DAB2IP through the EZH2-mediated H3K27me3 in 16-HBE cells exposed to CSE. These findings might suggest and support the connection between the habit to cigarette smoke (a risk factor for COPD), and the EZH2/ H3K27me3 and DAB2IP suppression in the airways of COPD patients. Moreover we speculate that, since GSK343 is a potent, selective and cell-active inhibitors of the methyltransferase EZH2^29^, its use might be able to down-regulate H3K27me3 activity in pathological conditions.

The characterization of *in vitro* models is crucial to the understanding of the distinct mechanisms implicated in the progression and invasion of lung cancer. However Polette M *et al*. describe that the 16HBE are not suitable to define the alteration of metastatic phenotype^42^. We studied the mechanisms implicated in the progression and invasion of lung cancer (apoptosis, invasion capacity and vimentin expression) due to CSE and EZH2-mediated, using A549 rather than 16HBE in *“in vitro”*. The analysis of transcription showed lower levels of DAB2IP mRNA expression in A549 cell line (adenocarcinoma phenotype) than epithelial cell line16HBE. DAB2IP mRNA was further reduced in the presence of CSE, while increased levels of EZH2 and H3K27me3 protein expression was observed. GSK343 pre-treatment restored the levels of DAB2IP mRNA and H3K27me3 in A549 than in 16HBE chronically exposed to CSE. Furthermore, GSK343 pretreatment of 16HBE and A549 cell line stimulated with CSE greatly increases the expression of DAB2IP compared to untreated cells. These data might provide the existence of epigenetic mechanisms different from H3K27 methylation as previously described^43^ (overall in A549 carcinoma cell line), by which EZH2 regulates the expression of DAB2IP onco-suppressor in epithelial cells exposed to cigarette smoke. On the other hand, the treatment of A549 cell line with GSK343 alone partially reduced the H3K27me3 levels in untreated cells and in the cells treated with CSE. Finally, we evaluated only the EZH2 and H3K27me3 proteins and not EZH2 and H3K27me3 mRNAs in cells exposed to cigarette smoke, since as previously showed^17^, this methodological approach seems to be adequate to define the epigenetic modifications of DAB2IP gene, by the action of methyl transferase EZH2 on H3K27 trimethylation in lysine 27.

Defects in apoptosis are implicated in both tumorigenesis and drug resistance, and these defects might be involved in chemotherapy failure in prostate cancer^43^. In the presence of EZH2 overexpression, EZH2 inhibitor suppress the migration partly origin from inhibition of cell proliferation, induction of apoptosis or interruption of cell cycle promoting mechanism of cellular protection in the lung^44,45^. We showed that CSE (10% and 20%) increased the Annexin V positive cells in stable cell line 16HBE and A549, and in these experimental conditions the cells AnnexinV positive further increased in the presence of GSK343 (the inhibitor of the methyltransferase EZH2). These data suggest that the inhibition of EZH2 activity by GSK343 might promote an apoptotic fate instead of a cancerous route in the epithelial cells. However, in accord with normal or tumor phenotype of the cell line, which makes the A549 more proliferative than the 16HBE, we showed higher values of cell apoptosis in normal epithelial cell line (16HBE) rather than in cancer cell line (A549). The findings lead us to consider that EZH2 might favor the proliferative processes playing a critical role in the structural alteration observed in the area of metaplasia present in the epithelium of the airways of COPD patients, independently to the phenomena of cell apoptosis generated by the cigarette smoke. However, further study might be necessary to better clarify the role of EZH2 in the control and regulation of the mechanism of cell apoptosis and proliferation in airway epithelial cells.

DAB2IP is transcriptionally down regulated in a variety of tumors and is involved in epithelial to mesenchymal transition (EMT) and prostate cancer metastasis^46^. We analyzed the vimentin expression and performed a *matrigel invasion assay* in A549 cells treated with CSE in the presence or absence of GSK343. We found that 14 days of CSE stimulation could induce the increase of vimentin expression and cell invasion, reduced by GSK343 treatment in A549 cell line exposed to CSE. In agreement with the fact that the 16HBE were not suitable to define the alteration of metastatic phenotype^42^, we did not observe alterations of vimentin expression in 16HBE stimulated with CSE (data not shown). These results have encouraged us to select A549 cell line to study the role of EZH2 on EMT. In this context, we identified the potential role of methyl-transferase EZH2 in the control of cell infiltration during the program of the potential malignant state of the cells, which involve EMT profiles by vimentin expression in epithelial cells (A549 cells). All together, our findings could open new perspectives on the relevance of the EZH2 on the regulation of DAB2IP expression in the airway inflammation. The habit of cigarette smoking alters the balance of the expression of these markers, promoting molecular events triggering epigenetic modification of DAB2IP oncogene, promoting the progression of inflammatory disease towards lung cancer.

## Conclusions

**Figure 10:**
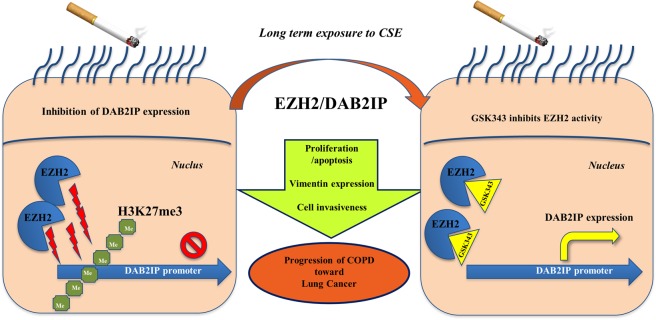
Summary of the study. The cellular and molecular mechanisms associated to EZH2 and DAB2IP regulation represent a potential link between chronic inflammation and COPD progression towards lung cancer. The habit to cigarette smoke affects the methyl transferase EZH2 activity, affecting a key mechanism in the control of DAB2IP onco-suppressor, cell apoptosis, vimentin expression, and cell invasiveness in bronchial epithelial cells of COPD patients. The use of EZH2 inhibitor (GSK343) could represent as innovative therapeutic target to prevent biomolecular transition of COPD toward lung cancer.

In conclusion, the cellular and molecular mechanisms associated to EZH2 and DAB2IP regulation represent a potential link between chronic inflammation and COPD progression towards lung cancer. This study demonstrates how the habit to cigarette smoke affects the methyl transferase EZH2 activity, which might have a key role in the control of onco-suppressor DAB2IP, cell apoptosis, vimentin expression, and cell invasiveness in bronchial epithelial cells of COPD patients. Our findings provide future perspectives for new pharmacological approach in the treatment of COPD patients, suggesting EZH2 as innovative therapeutic target to prevent biomolecular transition of COPD toward lung cancer. However, the descriptive nature of our data requires more insight into the cellular and molecular regulation of EZH2/DAB2IP balance in epithelium of the airway of subjects who smoke (Fig. 10).

## Supplementary information

Supplementary Information File
