# Supplementary material for: Cigarette smoke affects the onco-suppressor DAB2IP expression in bronchial epithelial cells of COPD patients
Source: Sci Rep. 2019 Oct 30;9:15682. doi: 10.1038/s41598-019-52179-5 (PMC6821751; doi:10.1038/s41598-019-52179-5)
Supplement: Supplementary file 1 — Supplementary Information File [file 41598_2019_52179_MOESM1_ESM.pdf]

Title: Cigarette smoke affects the onco-suppressor DAB2IP expression in bronchial epithelial cells of COPD patients.

Running title: EZH2 and DAB2IP in COPD**.**

GiuliaAnzalone[1], Giuseppe Arcoleo[1],Fabio Bucchieri[1,2], Angela MMontalbano[1], Roberto Marchese[3], Giusy D Albano[1],Caterina Di Sano[1], MonicaMoscato[1], Rosalia Gagliardo[1], Fabio LM Ricciardolo[4], MirellaProfita[1]

[1]Institute of Biomedicine and Molecular Immunology "A. Monroy" (IBIM), National Research Council of Italy (CNR), Palermo, Italy.

[2] Dipartimento di Biomedicina sperimentale e Neuroscienze Cliniche (BioNec), University of Palermo, Palermo, Italy

[3]InterventionalPulmonology Unit, La Maddalena Cancer Center, Palermo, Italy.

[4]Department of Clinical and Biological Sciences, University of Torino, Torino, Italy

Corresponding Author:

Mirella Profita, PhD

Institute of Biomedicine and Molecular Immunology "A. Monroy" (IBIM),

National Research Council of Italy (CNR), Palermo, Italy

Via Ugo La Malfa 153, 90146 Palermo, Italia.

E-mail: **profita@ibim.cnr.it**

Key words: Enhancer of zester homolog 2 (EZH2), disabled homolog 2 interacting protein gene (DAB2IP), Cigarette smoke, Chronic obstructive pulmonary disease (COPD), Lung cancer.

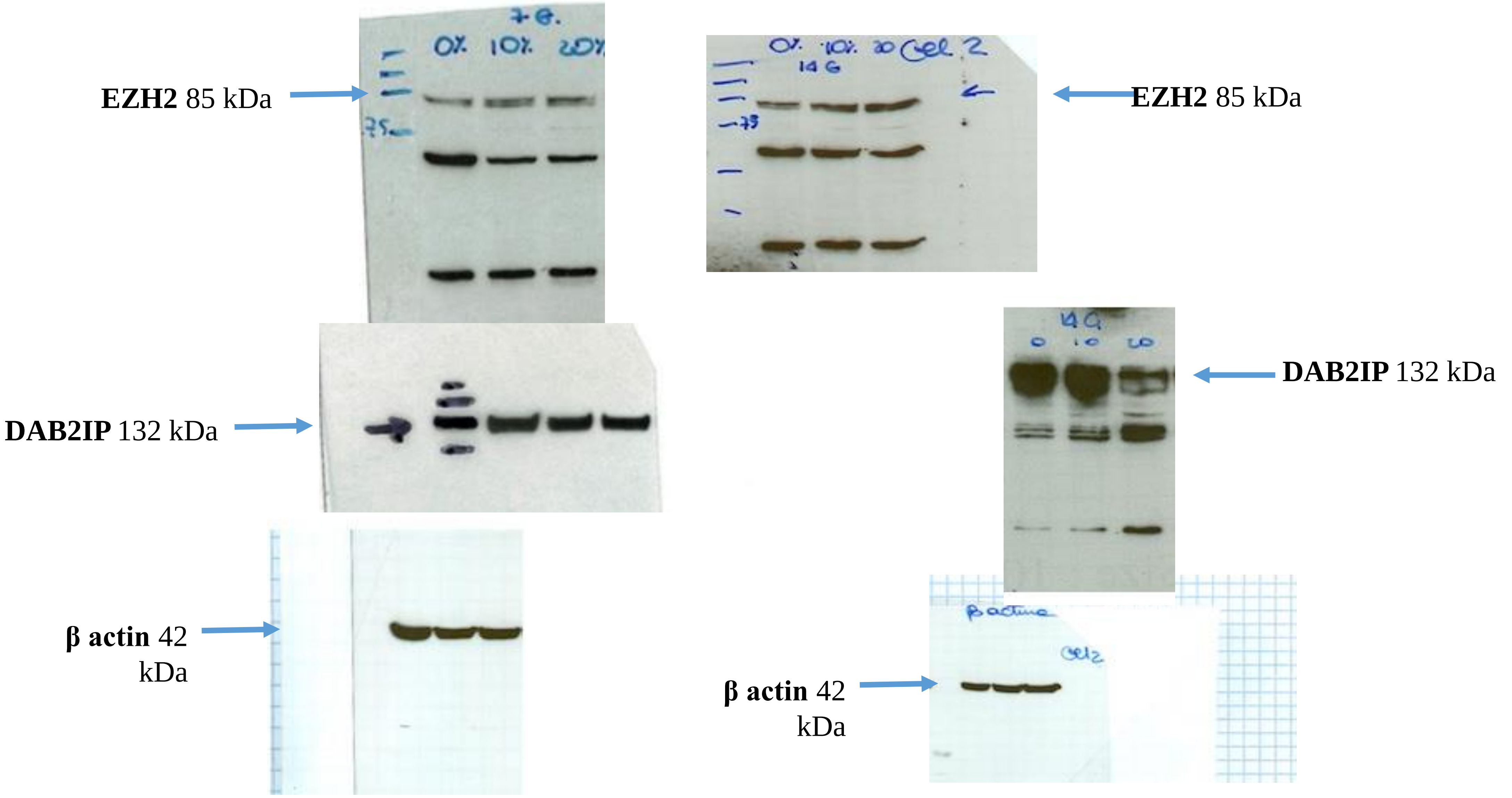

WB Figure 3 MS SRE-18-00417: Cigarette smoke affects the onco-suppressor DAB2IP expression in bronchial epithelial cells of COPD patients. GiuliaAnzalone[1], Giuseppe Arcoleo[1],Fabio Bucchieri[1,2], Angela MMontalbano[1], Roberto Marchese[3], Giusy D Albano[1],Caterina Di Sano[1], MonicaMoscato[1],Rosalia Gagliardo[1], Fabio LM Ricciardolo[4], MirellaProfita[1]

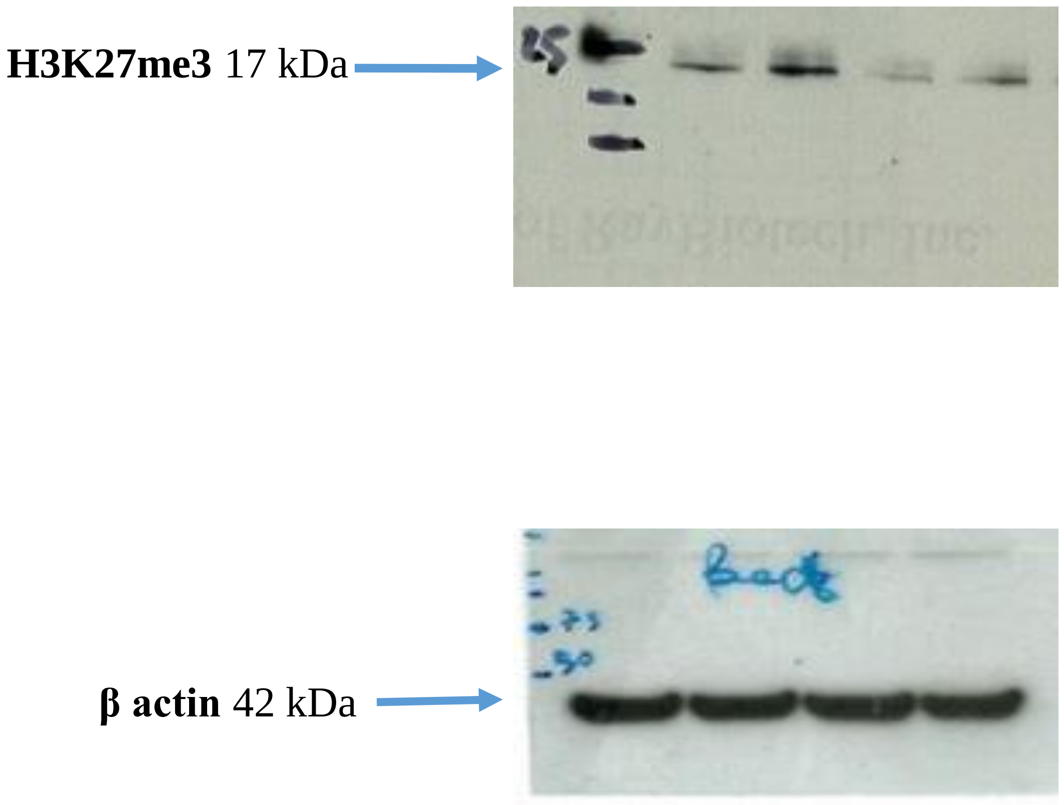

WB figure 4 MS SRE-18-00417: Cigarette smoke affects the onco-suppressor DAB2IP expression in bronchial epithelial cells of COPD patients. GiuliaAnzalone[1], Giuseppe Arcoleo[1],Fabio Bucchieri[1,2], Angela MMontalbano[1], Roberto Marchese[3], Giusy D Albano[1],Caterina Di Sano[1], MonicaMoscato[1],Rosalia Gagliardo[1], Fabio LM Ricciardolo[4], MirellaProfita[1]

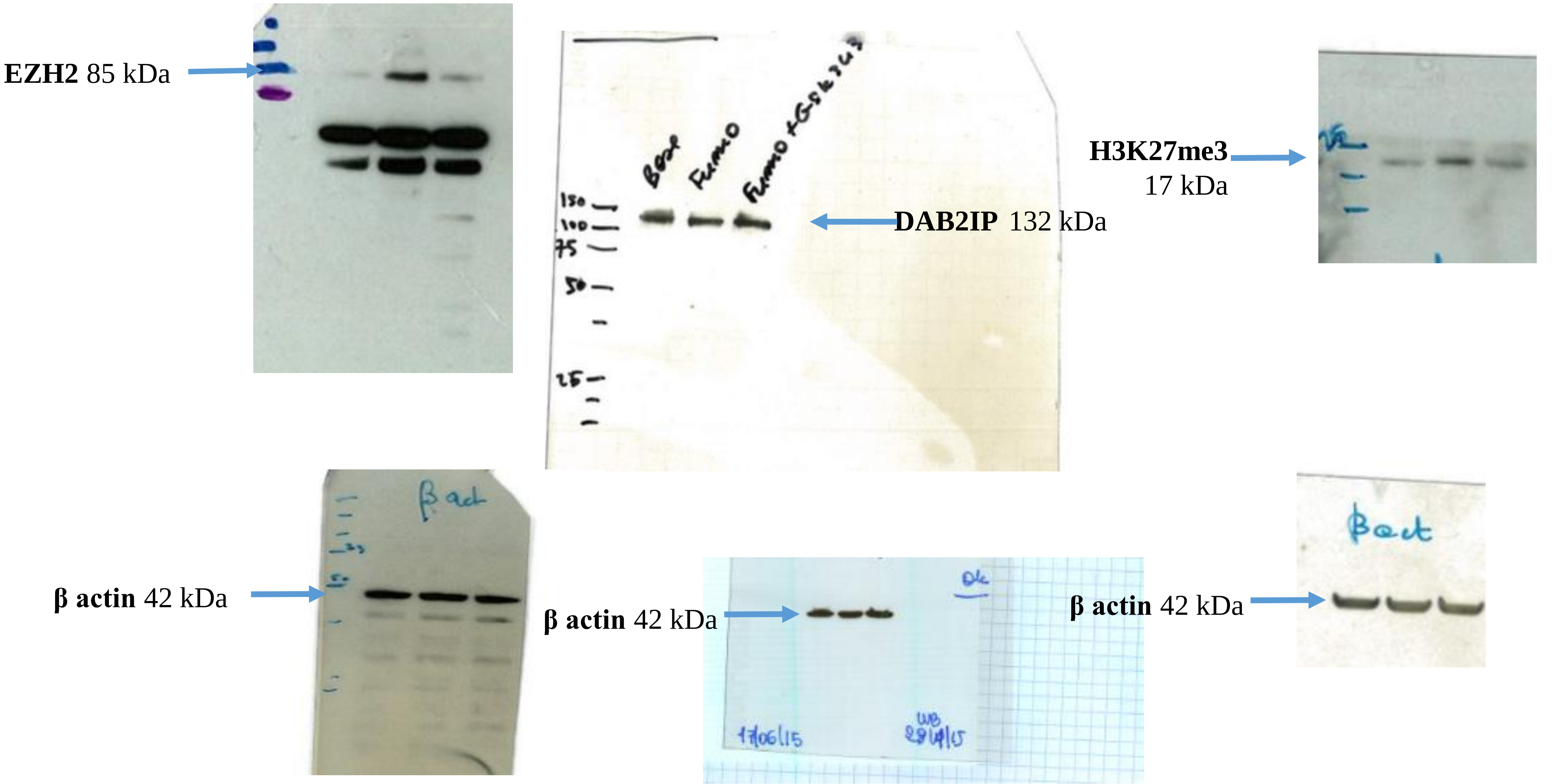

WB Figure 5 MS SRE-18-00417: Cigarette smoke affects the onco-suppressor DAB2IP expression in bronchial epithelial cells of COPD patients. GiuliaAnzalone[1], Giuseppe Arcoleo[1],Fabio Bucchieri[1,2], Angela MMontalbano[1], Roberto Marchese[3], Giusy D Albano[1],Caterina Di Sano[1], MonicaMoscato[1],Rosalia Gagliardo[1], Fabio LM Ricciardolo[4], Mirella Profita[1]

H3K27me3 17 kDa

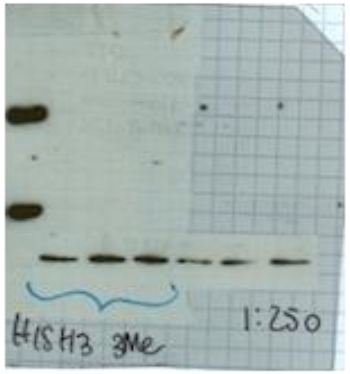

β actin 42 kDa

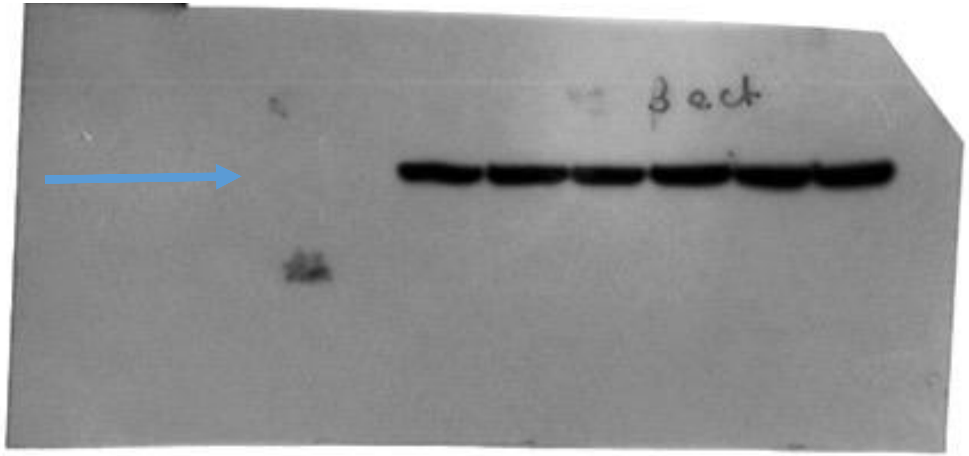

WB figure 6 MS SRE-18-00417: Cigarette smoke affects the onco-suppressor DAB2IP expression in bronchial epithelial cells of COPD patients. GiuliaAnzalone[1], Giuseppe Arcoleo[1],Fabio Bucchieri[1,2], Angela MMontalbano[1], Roberto Marchese[3], Giusy D Albano[1],Caterina Di Sano[1], MonicaMoscato[1],Rosalia Gagliardo[1], Fabio LM Ricciardolo[4], MirellaProfita[1]

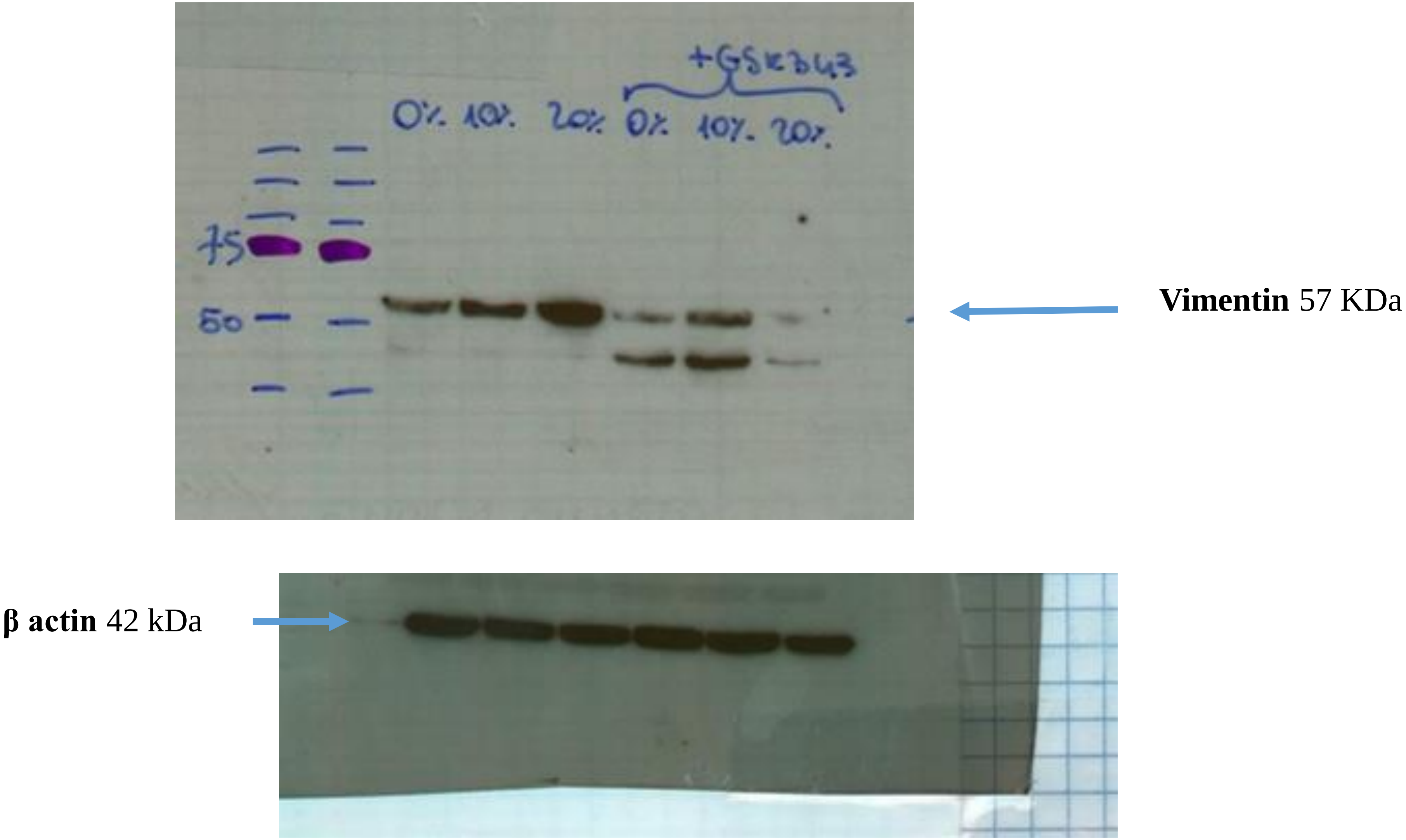

WB Figure 9 MS SRE-18-00417: Cigarette smoke affects the onco-suppressor DAB2IP expression in bronchial epithelial cells of COPD patients. GiuliaAnzalone[1], Giuseppe Arcoleo[1],Fabio Bucchieri[1,2], Angela MMontalbano[1], Roberto Marchese[3], Giusy D Albano[1],Caterina Di Sano[1], MonicaMoscato[1],Rosalia Gagliardo[1], Fabio LM Ricciardolo[4], MirellaProfita[1]
